# Genomic language model for predicting enhancers and their allele-specific activity in the human genome

**DOI:** 10.1093/bioinformatics/btag456

**Published:** 2026-09-10

**Authors:** Rekha Sathian, Pratik Dutta, Ferhat Ay, Ramana V Davuluri

**Affiliations:** Department of Biomedical Informatics, Stony Brook University, Stony Brook, NY 11794, United States; Department of Biomedical Informatics, Stony Brook University, Stony Brook, NY 11794, United States; Centers for Cancer Immunotherapy and Autoimmunity, La Jolla Institute for Immunology, La Jolla, CA 92037, United States; Bioinformatics and Systems Biology Graduate Program, University of California, San Diego, La Jolla, CA 92093, United States; Department of Pediatrics, University of California, San Diego, La Jolla, CA 92093, United States; Department of Biomedical Informatics, Stony Brook University, Stony Brook, NY 11794, United States

## Abstract

**Motivation:**

Predicting and deciphering the regulatory logic of enhancers remains a significant challenge due to their complex sequence features and the absence of consistent genetic or epigenetic signatures that distinguish them from other genomic regions. Existing machine learning methods capture nucleotide composition but often fail to model sequence context effectively.

**Results:**

We present DNABERT-Enhancer, a novel enhancer prediction method, by applying DNABERT pre-trained language model on the human genome. Using ENCODE registry of candidate cis-regulatory elements (cCREs), we curated a benchmark dataset, consisting of 21 926 enhancers of 201 bp length and 46 159 enhancers of 350 bp length, as positive instances. The best fine-tuned model achieved 88.05% accuracy and a Matthews correlation coefficient of 76% on an independent dataset. Genome-wide application identified 1 684 595 enhancer regions covering 26.65% of the human genome. By performing integrative analyses with DNABERT-based transcription factor models, we identify 2681 statistically significant loss-of-function and 1917 gain-of-function enhancer variants, which respectively alter the function of 1623 and 1247 ENCODE-cCRE enhancers. Similarly, we identify 4057 candidate *de novo* enhancers, created by 5464 gain-of-function variants. These genome-wide enhancer annotations and candidate genetic variants predicted by DNABERT-Enhancer provide valuable resources for genome interpretation in functional and clinical genomics studies.

**Availability and implementation:**

DNABERT-Enhancer is freely available at https://github.com/DavuluriLab/DNABERT-Enhancer; Trained model predictions can be explored interactively via the web application at https://dnabert-enhancer-datarepo.streamlit.app/. The fine-tuned models are archived and citable through Zenodo (https://doi.org/10.5281/zenodo.19157566).

## 1 Introduction

Eukaryotic enhancers are cis-acting gene regulatory elements, which enhance transcriptional activation, thereby shaping the characteristics and functions of cells, tissue, and organisms. These 100–1500 bp spanning genomic sequences constitute ∼30% of the noncoding part of the human genome, suggesting they are far more numerous than the approximately 20 000 protein-coding genes in the human genome (Pennacchio *et al.* 2013, Panigrahi and O’Malley 2021). Their functional capability hinges on their architecture, including binding affinities, transcription factor (TF) types, and underlying topology (Kurdistani 2012, Galupa *et al.* 2023). Disruption in the ideal function of enhancers due to genetic phenomena like enhancer hijacking, disruption of Topologically Associating Domain (TAD), epigenetic modulation, or structural variants can lead to genetic diseases like congenital disorders, cancers, and common complex diseases, collectively termed as enhanceropathies (Kurdistani 2012, Corradin and Scacheri 2014, Herz *et al.* 2014, Sur and Taipale 2016). genome-wide association studies (GWAS) have identified risk variants (Blackwood and Kadonaga 1998), mainly SNPs, in enhancer elements, highlighting their clinical relevance in therapeutic strategies (Kurdistani 2012, Nasser *et al.* 2021). Most of the disease-associated noncoding SNPs are known to affect enhancer function by biased allelic activity (Duan *et al.* 2023, Gao *et al.* 2025). Experimental screening methods, such as CRISPR-Cas9, self-transcribing active regulatory region sequencing (STARR-seq), and massively parallel reporter assays (MPRA) have emerged as powerful tools for assessing the regulatory activity of candidate SNPs (Pickar-Oliver and Gersbach 2019). However, these experimental methods are limited by their low throughput and laborious protocols (Ni *et al.* 2023, Safaeesirat *et al.* 2025). Therefore, advanced computational methods are in critical need for genome-wide analyses of enhancer affecting SNPs.

The multifaceted nature of enhancers—their varying lengths, ambiguous boundaries, orientation-independent function, and variable distance from associated promoters—complicates their computational identification and functional annotation (Benton *et al.* 2019, Smith *et al.* 2023). In addition, although general features of enhancers are known, there is no consensus on which features need to be present at what levels to declare a region as “enhancer”. Considering the profound biological and clinical significance of enhancers, the research community consistently endeavored to identify and better understand these noncoding regions (The ENCODE Project Consortium *et al.* 2020). While several experimental methods, such as ChIP-seq (Visel *et al.* 2009), ATAC-seq (Buenrostro *et al.* 2013), DNase-seq (Boyle *et al.* 2008), CAGE (Guerrini *et al.* 2022), GRO-seq (Lopes *et al.* 2017), and PRO-seq (Kwak *et al.* 2013) have been applied for genome-wide mapping of candidate enhancers, their precise identification comprehensively across many conditions and cell types has remained elusive. Recent advances in high throughput reporter assays (Kwasnieski *et al.* 2012, Melnikov *et al.* 2012, Arnold *et al.* 2013) have enabled the design and testing of synthetic enhancers, revealing how sequence composition dictates enhancer strength and specificity (King *et al.* 2020). This led to development of a flurry of ML-based predictive models for enhancer identification.

One of the most widely used approaches is the use of epigenetic information, e.g. ChromaGenSVM (Fernandez and Miranda-Saavedra 2012) uses histone marks, CSI-ANN (Firpi *et al.* 2010) utilizes the chromatin signatures, and RFECS (Rajagopal *et al.* 2013) integrates histone modification profiles. However, these methods often fail to learn the sequence context of the functional region itself. To address this, subsequent methods like iEnhancer-2L (Liu *et al.* 2016), iEnhancer-XG (Cai *et al.* 2021), iEnhancer-EL (Liu *et al.* 2018), and EnhancerPred (Jia and He 2016) focused on extracting sequence features or nucleotide composition, achieving improved accuracy. Furthermore, while some recent methods have applied convolutional neural network (CNN)-based deep learning approaches (Kim *et al.* 2016, Liu *et al.* 2016, Min *et al.* 2017, Nguyen *et al.* 2019), few others have used hybrid methods to leverage the advantages of two different model architectures, e.g. DEEP (Kleftogiannis *et al.* 2015), iEnhancer-DCLA (Liao *et al.* 2022), and BiRen (Yang *et al.* 2017). More recently, NLP based methods with an additional layer of classification algorithm were developed, like BERT-enhancer (Le *et al.* 2021) and iEnhancer-ELM (Li *et al.* 2023). The former method feeds the BERT-based features into a CNN, whereas the latter attaches a 2-layer perceptron network as the classifier to the pre-trained DNABERT model.

Treating DNA sequences like text, we earlier developed DNABERT language model for capturing DNA’s contextual and semantic information in the human genome (Ji *et al.* 2021), by adapting the Bidirectional Encoder Representations from Transformers (BERT) model that was also applied in many NLP tasks (Lee *et al.* 2020). DNABERT demonstrated top-tier performance in various downstream tasks including predicting promoters (DNABERT-Prom), splice sites (DNABERT-Splice), and TF binding regions. An updated version of DNABERT, DNABERT-2 (Zhou *et al.* 2023) was recently released for multiple species genomes. Based on the performance of DNABERT pre-trained model, we hypothesized that DNABERT could be fine-tuned to predict enhancer sequences by leveraging its attention mechanism to identify regions critical for enhancer function.

Here, the goal is to predict the allelic bias of SNPs within an input DNA sequence based on highly accurate fine-tuned models that can classify a random genomic sequence as enhancer or non-enhancer based on DNABERT embeddings. We curated a large collection of enhancer sequences, which include ENCODE data (The ENCODE Project Consortium 2012) available through SCREEN (The ENCODE Project Consortium *et al.* 2020), and developed a novel enhancer prediction method, called DNABERT-Enhancer. We demonstrated that DNABERT-Enhancer captured contextual information from the entire input sequence with an attention mechanism, achieving superior performance on both independently set aside test data and genome-wide predictions. DNABERT-Enhancer is then applied for predicting functional noncoding SNPs that overlap with the SCREEN enhancers in the human genome. Finally, DNABERT TF fine-tuned models were applied to predict candidate noncoding genomic variants that disrupt TF target regions within the enhancers.

## 2 Materials and methods

### 2.1 Enhancer datasets

Enhancers were curated from the Registry of candidate cis-Regulatory Elements (cCREs) anchored on DNase I hypersensitive sites (DHSs) ranging from 150 to 350 bp and annotated using combinatorial chromatin signatures (H3K27ac, H3K4me3, and CTCF) from experimental datasets generated by the ENCODE Project Consortium. The ENCODE cCREs v3 dataset (GRCh38) contains 961 227 annotated enhancers (The ENCODE Project Consortium *et al.* 2020). For cell-type-specific modeling, enhancers active in neural progenitor cells and SK-N-SH (brain lineage), and breast epithelium cells and MCF-7 (breast lineage) were selected.

Additional enhancer regions from experimental and predicted enhancer databases were collected to assist negative instance generation. Negative sequences were sampled from non-enhancer regions of the human reference genome (GRCh38) while excluding annotated enhancers. This strategy was applied across both the global and cell-type-specific models. Positive and negative sequences were fragmented into fixed lengths of 201 and 350 bp to generate two datasets, which were balanced using a 1:1 enhancer to non-enhancer ratio.

### 2.2 Fine-tuning of DNABERT-Enhancer

#### 2.2.1 DNABERT-Enhancer model architecture

**Figure 1:**
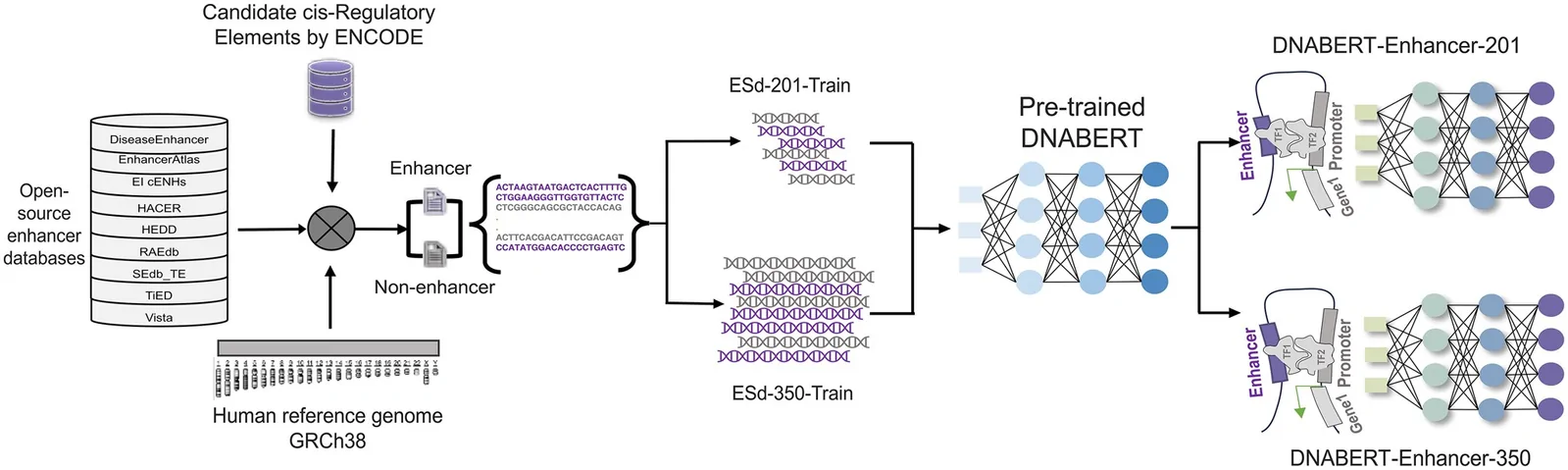
Overview of the DNABERT-Enhancer model development workflow. Candidate cis-regulatory elements (cCREs) from ENCODE were used as positive sequences (enhancers). To generate negative (non-enhancer) sequences from the human reference genome (GRCh38), cCREs, and the annotated enhancers from open-source databases were excluded. The resulting enhancer (positive) and non-enhancer (negative) sequences were partitioned into training datasets of 201 and 350 bp lengths (ES4-201-Train and ES4-350-Train). These sequences were tokenized and input to the pre-trained DNABERT language model, which was subsequently fine-tuned to build two variant-length enhancer prediction models, DNABERT-Enhancer-201 and DNABERT-Enhancer-350. The fine-tuned models learn sequence context and regulatory patterns to distinguish enhancer from non-enhancer regions for genome-wide enhancer prediction.

We developed two models—DNABERT-Enhancer-201 and DNABERT-Enhancer-350—to predict enhancer regions from genomic sequences of length 201 and 350 bp, respectively (Fig. 1). Both models were fine-tuned from the DNABERT base architecture, which follows the BERT-base configuration: 12 transformer encoder layers, 12 self-attention heads per layer, a hidden size of 768, and approximately 110 million parameters. DNABERT was originally pre-trained on the human reference genome using *k*-mer tokenization. In our framework, the input genome sequences were tokenized into overlapping 6-mers and converted into embedding representations. Embeddings were processed through stacked self-attention layers to capture contextual dependencies within the sequence. The final hidden representations were passed to a classification head to generate a probability score indicating the likelihood of a sequence being an enhancer. We applied different decision thresholds to convert predicted probabilities into binary labels (y ∈ {1,0}) to test model robustness. Additionally, we evaluated the models using threshold-independent metrics, including ROC and precision–recall curves.

#### 2.2.2 Training and testing of DNABERT-enhancer models

Each model was trained on 80% of enhancer sequences—(ESd-201-Train, ∼17 500 enhancers and ESd-350-Train, ∼36 900 enhancers), with an equal number of non-enhancers randomly sampled to maintain class balance. The best trained models were tested on the remaining 20% of the sequences that were held out (ESd-201-Test, ∼4300 each in enhancer and non-enhancer classes and ESd-350-Test, ∼9200 each in enhancer and non-enhancer classes). To optimize performance and mitigate overfitting, we applied several standard deep learning strategies, including dropout regularization, early stopping based on validation loss, and data augmentation techniques such as random sequence trimming and shuffling. Hyperparameter tuning was conducted across a range of learning rates (1 × 10^−3^ to 3 × 10^−6^), warm-up steps (0.1 and 0.2), and weight decay values (0.0001, 0.001, 0.005, 0.01, and 0.02), while maintaining a consistent dropout throughout. The training process was performed on eight NVIDIA A40 GPUs with each hyperparameter setting requiring approximately 4–5 h of training time.

#### 2.2.3 Genome-wide application of DNABERT-enhancer model

To assess genome-wide performance, GRCh38 sequences were scanned using sliding windows of 350 bp (step size 50 bp). Each window was assigned an enhancer prediction probability by the model, and windows with probability ≥0.5 were considered enhancer. Consecutive windows were evaluated in genomic order and merged to define continuous enhancer regions. To account for the increased overlap introduced by the smaller step size and to avoid fragmentation due to short fluctuations in prediction scores, brief stretches of low-probability windows were tolerated during region merging, while sustained low-probability segments were used to terminate enhancer regions (see Supplementary Methods, available as supplementary data at *Bioinformatics* online). Predicted enhancers were evaluated by overlap with known enhancer databases at multiple cutoffs, and statistical significance was assessed via permutation.

### 2.3 Variant effect prediction

To evaluate the functional impact of genetic variants within SCREEN enhancers, we analyzed dbSNP short variants (GRCh38) by substituting variant alleles into enhancer sequences and computing predictions using DNABERT-Enhancer-350. Variants in TF target regions within enhancers were assessed using DeepVRegulome, a deep-learning framework trained on ChIP-seq data for TFs and histone marks. Gain-of-function (GOF) and loss-of-function (LOF) regulatory variants were quantified based on changes in predicted enhancer probability (detailed in Supplementary Methods, available as supplementary data at *Bioinformatics* online), with thresholds applied to identify variants with strong predicted regulatory effects. Candidate variants were cross-referenced with known functional annotations from ClinVar, GWAS catalogs, and high-confidence cis-eQTL datasets.

## 3 Results

### 3.1 Benchmark enhancer dataset

**Figure 2:**
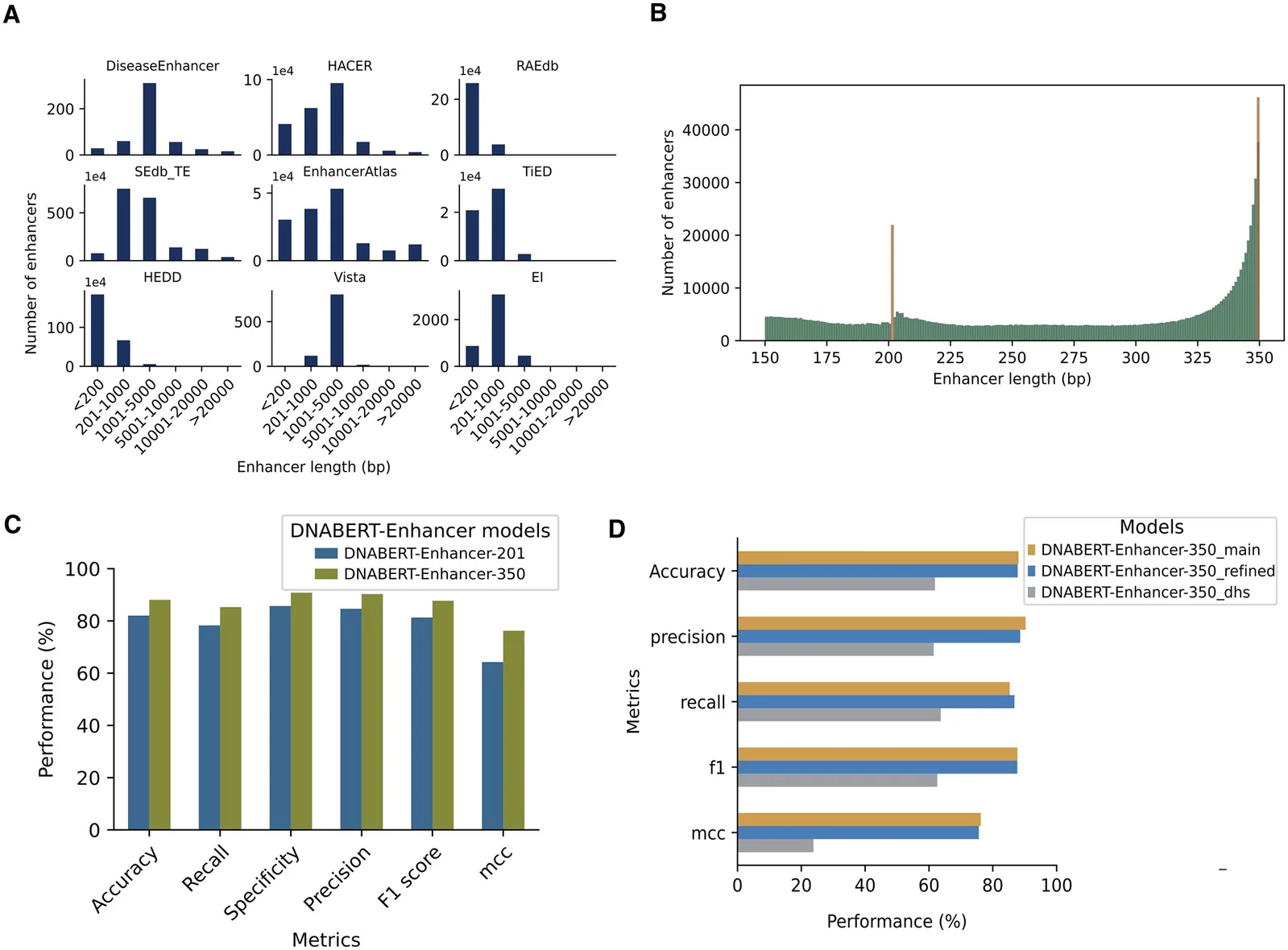
Dataset composition and performance evaluation of DNABERT-Enhancer models. (A) Length distribution of enhancers from publicly available databases namely, DiseaseEnhancer, HACER, RAEdb (top row), typical elements from SEdb (SEdb_TE), EnhancerAtlas, TiED (middle row) and HEDD, Vista, EI (bottom row). (B) Length distribution of enhancer regions downloaded from the Registry of candidate cis-Regulatory Elements (cCREs) in ENCODE encyclopedia, showing the peaks at 201 and 350 bp. (C) Performance metrices namely accuracy, precision, recall, *F*1 score, and MCC (bars from left to right) of the two DNABERT-Enhancer models: DNABERT-Enhancer-201 trained on ESd-201-Train and tested on ESd-201-Test data and DNABERT-Enhancer-350 trained on ESd-350-Train and tested on ESd-350-Test data. (D) DNABERT-Enhancer-350 main model compared to model trained with refined non-enhancer data and other DNase hypersensitivity sites from ENCODE cCREs.

Enhancer datasets in many published methods use fixed-length sequences (∼200 bp), approximating nucleosome plus linker DNA. In contrast, curated enhancers in public databases vary widely, spanning from a few hundred to over a thousand base pairs (Fig. 2A). Based on the ENCODE enhancer length distribution, which shows prominent peaks at 201 and 350 bp (Fig. 2B), we curated two benchmark sets: Set 1 with 21 926 enhancers (∼2%) of 201 bp and Set 2 with 46 159 enhancers (∼5%) of 350 bp as positive instances. The larger number of 350 bp enhancers reflects the inherent length distribution of cCRE elements, which shows a higher peak at 350 bp than at 201 bp.

For each set, equal numbers of non-enhancer regions of matching lengths (201 and 350 bp) were sampled as negative instances to avoid class imbalance. Comparison of genomic distances from transcription start sites (TSS) showed that negatives were generally farther from TSS than positives, although their distributions substantially overlapped (Fig. 1, available as supplementary data at *Bioinformatics* online), indicating that classification is not driven solely by promoter proximity. These datasets were used for model fine-tuning and benchmarking.

### 3.2 DNABERT-Enhancer accurately predicts enhancer regions

We fine-tuned two enhancer prediction models for 201 and 350 bp sequences. DNABERT-Enhancer-350 achieved 88.05% accuracy (MCC 76.22%), while DNABERT-Enhancer-201 reached 82.04% accuracy (MCC 64.27%) (Fig. 2C). We evaluated model performance across multiple thresholds to test prediction robustness and threshold impact (see Supplementary Results, available as supplementary data at *Bioinformatics* online; Fig. 2A, available as supplementary data at *Bioinformatics* online). Hyperparameter tuning optimized performance while limiting overfitting. DNABERT-Enhancer performed best on 350 bp data with a 3e−5 learning rate, 0.2 warm-up, and 0.0001 weight decay, whereas the 201 bp model performed best with a 0.001 learning rate, 0.1 warm-up, and 0.01 weight decay. Figure 2B–D, available as supplementary data at *Bioinformatics* online, demonstrates the progressive enhancement of our best model’s performance with each step, as evidenced by the increasing trends in accuracy, precision, recall, *F*1 scores, and MCC at minimal train loss. To assess the robustness of the model with respect to background sequence selection, we trained additional DNABERT-Enhancer-350 models using alternative negative sets and the results are described in Supplementary Results, available as supplementary data at *Bioinformatics* online (Fig. 2D).

### 3.3 DNABERT-Enhancer generalizes across and within cell-type-specific enhancer contexts

To further examine whether enhancer sequence features are predominantly shared across cellular contexts or enriched in a context-dependent manner, we constructed cell-type-specific models in addition to the global sequence-based model. The cell-type-specific models were trained independently using enhancers active in individual cellular contexts by selecting four representative cell types belonging two tissue lineages: neural progenitor cells and SK-N-SH (brain lineage), and breast epithelium cells and MCF-7 (breast lineage).

All cell-type-specific models achieved high predictive performance (Table 1). Specifically, the neural progenitor cell model achieved an accuracy of 89.83%, the SK-N-SH model 91.42%, the breast epithelium model 90.88%, and the MCF-7 model 90.94%. The comparable performance observed across distinct cellular contexts indicates that enhancer-associated sequence determinants are robust and learnable within individual lineages. Importantly, the strong performance of the global model, together with the high accuracy of the cell-type-specific models, suggests that enhancer architecture comprises both broadly shared regulatory sequence features and context-enriched components. The global model captures sequence patterns associated with general enhancer potential, whereas the cell-type-specific models likely emphasize lineage-restricted regulatory grammar.

**Table 1:** Summary of datasets and performance of cell-type-specific models from two tissue lineages.

Tissue-lineage	Cell-type-specific DNABERT-Enhancer models	Data_size (enhancer + non-enhancer)	Accuracy (%)	Precision (%)	Recall (%)	*F*1 score (%)	MCC (%)
Brain	Neural progenitor cells	30 729 + 30 729	89.83	91.27	88.09	89.65	79.71
	SK-N-SH	26 520 + 26 520	91.42	91.28	91.59	91.44	82.84
Breast	Breast epithelium	45 147 + 45 147	90.88	90.70	91.10	90.90	81.76
	MCF-7	35 421 + 35 421	90.95	91.31	90.50	90.91	81.89

### 3.4 DNABERT-Enhancer fine-tuned models outperform baseline classifiers

We validated the performance of DNABERT-Enhancer by comparing the efficiency of both our models to several traditionally used baseline methods namely random forest classifier (Breiman 2001), *K*-nearest neighbor (KNN) (Mucherino 2009), support vector classifier (SVC), Gaussian Naive Bayes, AdaBoost classifier, and multi-layer perceptron (MLP). Compared to deep, transformer-based neural network like BERT which learns high-level representations from raw text, the baseline models often require feature engineering, especially for text data, to learn. For a fair comparison, here we used the embeddings from DNABERT to represent the input sequences thereby facilitating the classification tasks and the best hyperparameters identified for each method are reported in Table 1, available as supplementary data at *Bioinformatics* online.

Evaluation metrics confirm that DNABERT-Enhancer achieved superior performance compared to all benchmarks on both the test datasets, ESd-201-Test and ESd-350-Test (Table 2). Though KNN classifier observed to have a higher precision (95.4%), the recall was the lowest (7.64%) across all models indicating that the KNN model overlooks or miss out the true positive values when predicting high-confidence enhancer regions. Both KNN and Gaussian Naive Bayes yielded weaker *F*1-scores and MCCs. When extending the sequence length from 201 to 350 bp, baseline models consistently improved, showing that longer sequence contexts enhance classification accuracy. Despite this gain, low MCC values of baseline models across both datasets underscored a weak correlation between predicted and true labels.

**Table 2:** Comparison between DNABERT-Enhancer and the baseline methods.^a^

Models	Model performance on ESd-201-Test					Model performance on ESd-350-Test				
	Accuracy (%)	Precision (%)	Recall (%)	*F*1 score (%)	MCC (%)	Accuracy (%)	Precision (%)	Recall (%)	*F*1 score (%)	MCC (%)
Random forest	74.74	75.36	73.51	74.42	49.49	78.12	79.49	75.81	77.61	56.31
KNN	57.1	82.01	18.19	29.78	22.62	53.63	**95.4**	7.64	14.14	18.54
SVC	73.47	73.56	73.28	73.42	46.95	78.07	77.47	79.15	78.3	56.14
Gaussian Naive Bayes	69.56	71.2	65.71	68.34	39.24	72.58	74.42	68.83	71.51	45.3
AdaBoost Classifier	75.73	75.4	76.38	75.89	51.46	77.9	77.8	78.07	77.93	55.8
MLP	69.24	68.99	69.9	69.45	38.49	77.2	76.84	77.88	77.36	54.41
DNABERT-Enhancer	**82.04**	**84.64**	**78.29**	**81.3**	**64.27**	**88.05**	90.27	**85.29**	**87.71**	**76.22**

a KNN (*K*-nearest neighbor), support vector classifier (SVC), and multi-layer perceptron (MLP). Bold values indicate the highest performance for each evaluation metric in the corresponding column.

### 3.5 DNABERT-Enhancer outperforms recent enhancer prediction methods

**Figure 3:**
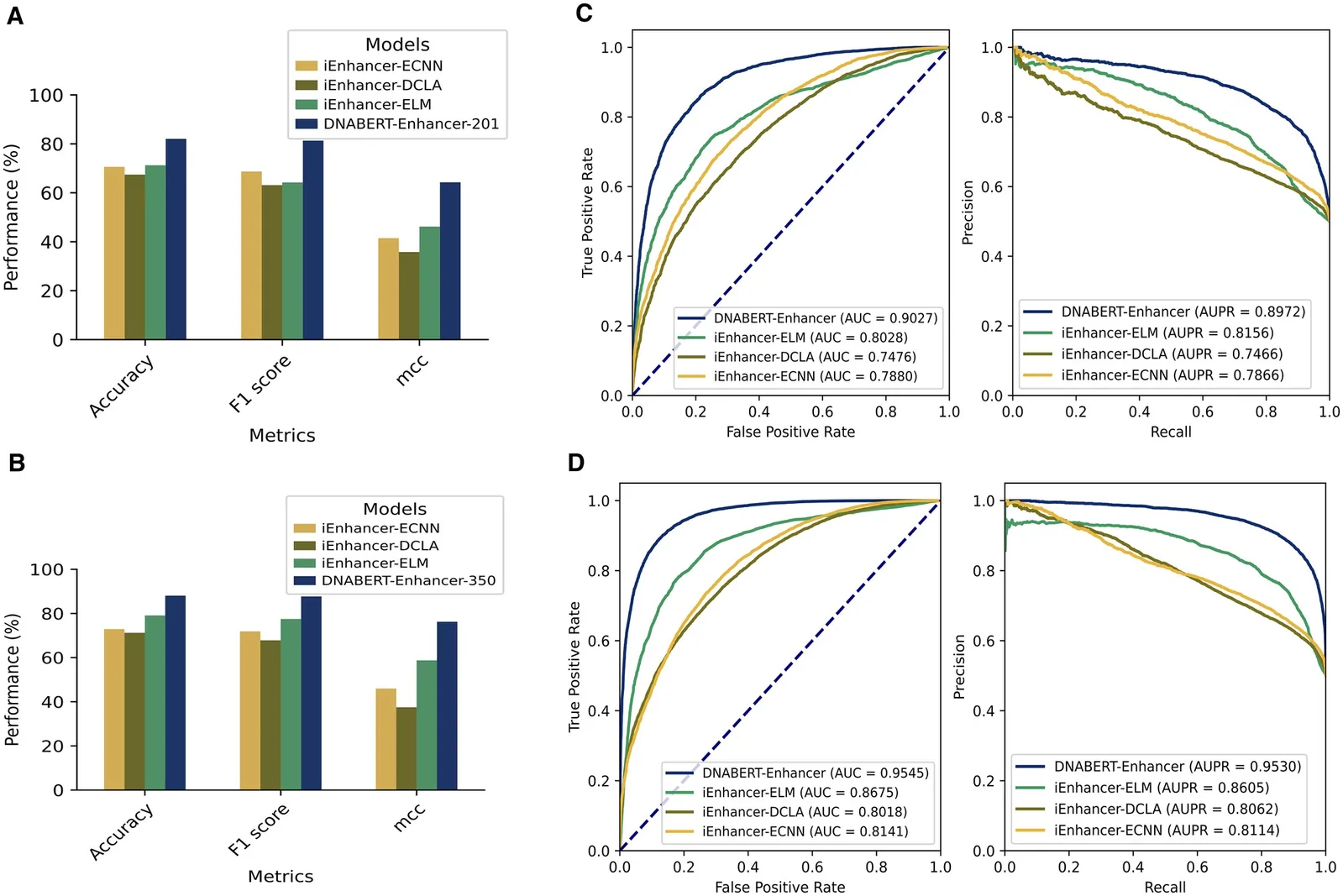
Comparison of DNABERT-Enhancer to the state-of-the-art methods namely, iEnhancer-ELM, iEnhancer-DCLA, and iEnhancer-ECNN. On left is the performance metrices accuracy (left), *F*1 score (middle), and MCC (right) of the models predicted on (A) ESd-201-Test and (B) ESd-350-Test. On the right is the ROC curve and precision–recall (PR) curve of the models predicted on (C) ESd-201-Test and (D) ESd-350-Test.

To benchmark DNABERT-Enhancer, we compared the performance of both models to the current state-of-the-art methods namely, iEnhancer-ELM (Li *et al.* 2023), iEnhancer-DCLA (Liao *et al.* 2022), and iEnhancer-ECNN (Nguyen *et al.* 2019). All models were evaluated on the same benchmark datasets using their publicly available implementations with default parameters. DNABERT-enhancer model outperformed the rest of the methods on ESd-350-Test data in all performance metrics. On ESd-201-Test data, our model exhibited high performance in all the performance metrics, except specificity (Table 2, available as supplementary data at *Bioinformatics* online). Though iEnhancer-ELM was observed to have a higher specificity (90.8%) in classifying 201 bp sequences, our model showed a significantly improved accuracy and *F*1 score compared to iEnhancer-ELM (Fig. 3A and B).

A noteworthy observation was the low MCC score of other models compared to our model. In a binary classification, MCC has proved to be the best evaluation metric, even over *F*1 score and accuracy as it considers all the four categorical values in a confusion matrix (Chicco and Jurman 2020). Thus, our model has demonstrated superior performance in discriminating enhancers from non-enhancer regions, regardless of the sequence length (Fig. 3C and D).

### 3.6 DNABERT-Enhancer outperforms recent enhancer prediction methods

To further contextualize the performance of DNABERT-Enhancer among recent genomic foundation models, we compared it against the nucleotide transformer (NT) (Dalla-Torre *et al.* 2025), a family of large-scale transformer models pre-trained on DNA sequences. We selected the NT 500M Human Reference model, which was pre-trained on the human reference genome, and fine-tuned it on our benchmark enhancer dataset using the same training and evaluation protocol applied to DNABERT-Enhancer (see Supplementary Methods, available as supplementary data at *Bioinformatics* online).

On the ESd-201-Test dataset, DNABERT-Enhancer achieved higher accuracy (82.04% versus 80.71%), precision (84.64% versus 79.55%), *F*1 score (81.30% versus 81.08%), and MCC (64.27% versus 61.47%) compared to the NT model, although NT showed slightly higher recall (82.67% versus 78.29%). Similarly, on the ESd-350-Test dataset, DNABERT-Enhancer achieved marginally better performance, with an accuracy of 88.05% and MCC of 76.22%, compared to 87.33% accuracy and 74.81% MCC for the NT model (Table 3). Despite its smaller size (∼110M versus ∼500M parameters), DNABERT-Enhancer achieved comparable or slightly better performance than NT across most metrics. This indicates that overlapping *k*-mer tokenization effectively captures enhancer regulatory grammar without requiring larger model capacity. Its compact architecture also enables more efficient fine-tuning and inference, supporting genome-wide applications such as the variant effect analyses in Section 3.8.

**Table 3:** Comparison between DNABERT-Enhancer and the nucleotide transformer (NT).

Models	Model performance on ESd-201-Test					Model performance on ESd-350-Test				
	Accuracy (%)	Precision (%)	Recall (%)	*F*1 score (%)	MCC (%)	Accuracy (%)	Precision (%)	Recall (%)	*F*1 score (%)	MCC (%)
Nucleotide transformer (NT)	80.71	79.55	**82.67**	81.08	61.47	87.33	89.94	84.06	86.90	74.81
DNABERT-Enhancer	**82.04**	**84.64**	78.29	**81.3**	**64.27**	**88.05**	**90.27**	**85.29**	**87.71**	**76.22**

Bold values indicate the highest performance for each evaluation metric in the corresponding column.

### 3.7 DNABERT-Enhancer successfully captures the known enhancers in the human genome

The results observed so far showcased the ability of DNABERT-Enhancer in accurately predicting the labeled sequences. However, to evaluate the genome-wide efficacy of our model in capturing the enhancer elements of varying lengths, we performed prediction on the whole human genome. We applied our best model, DNABERT-Enhancer-350 on a total of 59 381 268 subsequences of 350 bp length from the entire human genome. After merging overlapping sequences, we identified 1 684 595 distinct enhancer regions. These regions cover 26.65% of the human genome in base pair, including Chr X and Chr Y (Table 3, available as supplementary data at *Bioinformatics* online). These predicted enhancer regions were further validated by comparing them with the enhancer regions reported in open-source databases.

**Figure 4:**
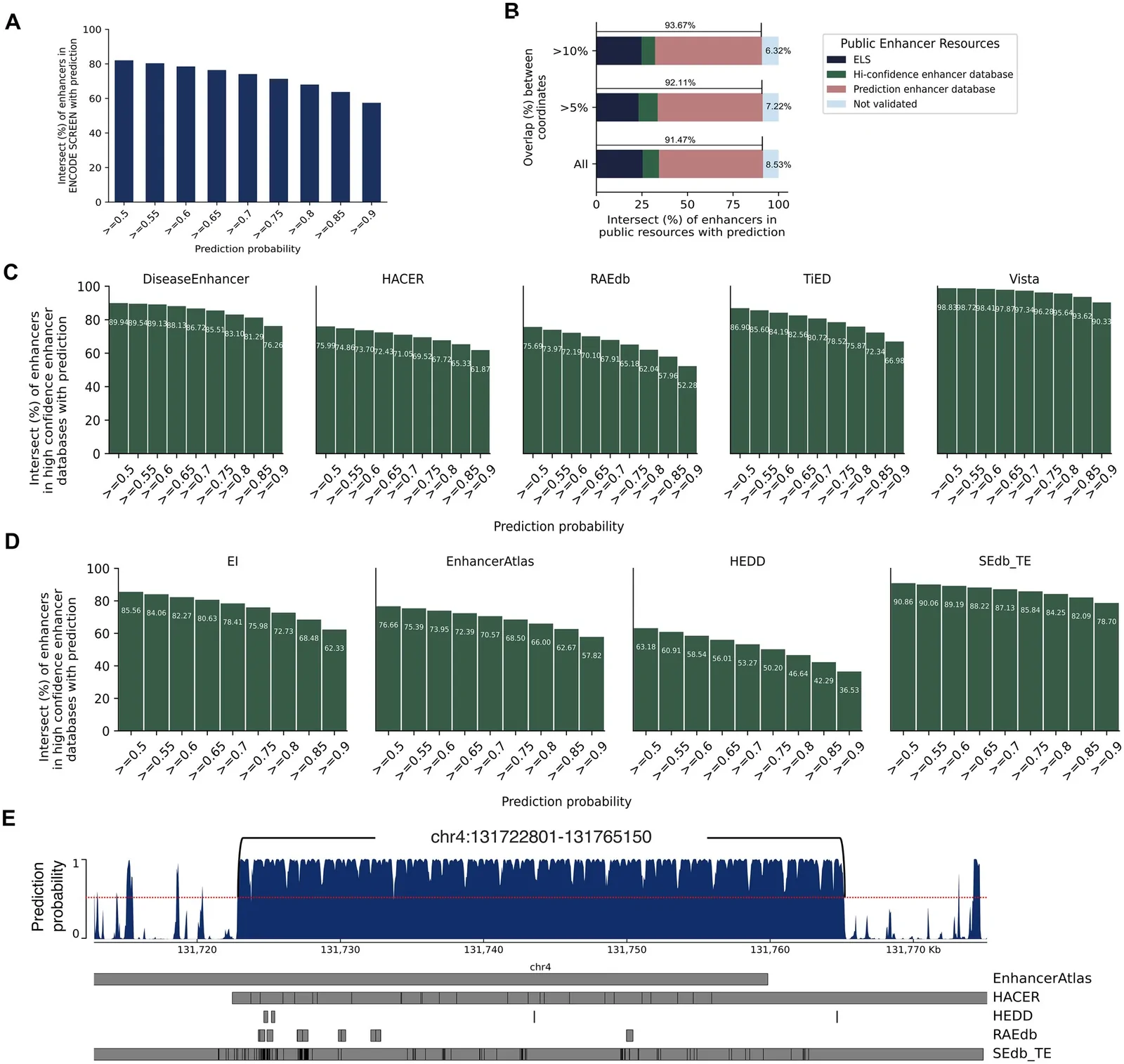
Prediction of DNABERT-Enhancer (350 bp) on whole human genome. (A) Intersection of predicted enhancers with enhancers in ENCODE SCREEN. (B) Proportion of predicted enhancers validated using public data sources. Intersection of predicted enhancers with (C) databases with high-confidence enhancer and (D) databases catalogued with predicted enhancers at different prediction probability cutoffs. (E) Depicting an enhancer region with length >40 kb, predicted by DNABERT-Enhancer, and the overlap of the region with multiple enhancer regions reported in five different open-source databases namely EnhancerAtlas, HACER, HEDD, RAEdb, and typical enhancers in SEdb (SEdb_TE).

Since our model was trained on ∼5.6% of the enhancer regions in the ENCODE SCREEN database, we compared the genome-wide prediction to the entire enhancer regions downloaded. About 25.44% of our predicted enhancers mapped to 788 686 enhancer regions in ENCODE SCREEN, which accounts for ∼82.05% of the entire collection (Fig. 4A and Table 4, available as supplementary data at *Bioinformatics* online). The remaining ∼17.95% of the SCREEN collection that were not predicted in the genome-wide analysis are shorter enhancer sequences, with a length less than 350 bp (Fig 3A, available as supplementary data at *Bioinformatics* online). Subsequently, we evaluated the remaining DNABERT-Enhancer predicted enhancer regions (∼74%, #1 256 106) which were not present in the SCREEN data using other public data sources for enhancer annotations (see Data Sources in Supplementary Methods, available as supplementary data at *Bioinformatics* online). During our initial assessment of these databases, we observed that some exhibited disproportionately high genomic coverage, and a considerable number of their enhancer annotations overlapped with protein-coding exonic regions. To avoid potential confounding effects in our validation process, we excluded all enhancer regions from these databases that overlapped with protein-coding exons prior to performing the overlap analysis. We found that 1 112 346 of 1 256 106 predictions (Fig. 4B and Fig. 3C, available as supplementary data at *Bioinformatics* online) intersect with enhancers from at least one of these databases, suggesting our method’s ability to capture potential enhancers beyond its training data.

Further, by comparing the genome-wide enhancer predictions with publicly available data sources (Tables 5 and 6, available as supplementary data at *Bioinformatics* online), we found that most of the predicted enhancers were represented in these databases (Fig. 4C and D). Notably, DNABERT-Enhancer-350 successfully identified approximately 98.83% of the 941 high-confidence enhancer sequences curated by comparative genomics and validated experimentally in the VISTA Enhancer Browser. Similarly, ∼76.66% of enhancer regions annotated in EnhancerAtlas—an interactive database of consensus enhancers derived through an unsupervised learning approach using diverse experimental datasets—overlapped with ∼54.98% of our model’s predictions. EnhancerAtlas contains approximately 60 000 enhancer regions longer than 5 kilobases (kb), and around 971 000 enhancer regions predicted by our model intersected with these large segments, highlighting DNABERT-Enhancer’s ability to capture components of super-enhancer regions. Additionally, 90.86% of the typical enhancer elements reported in SEdb (SEdb_TE) overlapped with 76.76% of our predicted enhancer regions, further demonstrating the model’s robustness in identifying functionally relevant enhancer elements. Permutation-based significance testing of these overlaps is described in the Supplementary Results, available as supplementary data at *Bioinformatics* online (Table 7, available as supplementary data at *Bioinformatics* online).

Due to the complicated positioning and orientation of enhancers with respect to their target genes and promoters, identification of a generalized sequence code for enhancers is challenging. The attention regions learned by DNABERT-Enhancer do not have any positional preferences within the enhancer regions (Fig. 3B, available as supplementary data at *Bioinformatics* online), rather are distributed in different locations of the predicted regions. For the same reason, the overlap of coordinates between the prediction result and the reported enhancers were expected to be partial. Moreover, our approach of identifying enhancer regions by tolerating brief dips within regions observed elements of varying length (Table 8, available as supplementary data at *Bioinformatics* online) with four elements that are larger than 40 kilobases(kb). Further, we observed that these long enhancer regions overlap with different enhancer regions reported in the open-source databases (Table 9, available as supplementary data at *Bioinformatics* online). Figure 4E depicts one such region in Chromosome 4 (chr4:131722801–131765150) of length 42 350 bp that mapped to varying length enhancer regions reported in five different data sources. The two step process of DNABERT-enhancer prediction on sliding window sequences and merging of overlapping sequences effectively predicted enhancer regions of varying lengths across the human genome.

### 3.8 DNABERT-Enhancer predicts candidate genetic variant effects in SCREEN enhancer regions

To systematically identify candidate regulatory variants with potential functional impact, we screened the 990 million short variants catalogued in dbSNP (Sherry *et al.* 2001) and mapped them to a defined subset of SCREEN enhancers (350 bp; ∼6% of total SCREEN regions). We reevaluated enhancer sequences using the DNABERT-Enhancer-350 model by substituting alternate alleles at variant positions. This analysis revealed 62 340 variants predicted to disrupt enhancer activity resulting in LOF effects across 5548 SCREEN enhancers (Table 10, available as supplementary data at *Bioinformatics* online). These results demonstrate widespread allele-specific sensitivity of enhancer activity to noncoding variation and provide a prioritized set of candidate regulatory variants.

To investigate potential mechanistic consequences, we assessed TF binding disruption using 458 highly accurate TF prediction DNABERT models from DeepVRegulome (each with ≥85% accuracy, trained on ENCODE ChIP-seq profiles). Comparative analysis between reference and alternate alleles identified 15 586 variants predicted to disrupt TF binding sites, affecting 342 TFs across 3066 enhancers (Table 11, available as supplementary data at *Bioinformatics* online). These results provide a basis for predicted LOF effects through disruption of regulatory protein–DNA interactions. Likewise, we identified 52 453 variants predicted to cause GOF effects in 2852 regions that were predicted as non-enhancers with reference allele. These variants increased the predicted enhancer probability upon substitution of the alternate allele, suggesting that certain mutations may create or strengthen enhancer activity *de novo*.

By applying a 5% empirical false discovery rate (FDR) threshold, we identified 2681 LOF SNPs (LOR > 2.65; Table 12, available as supplementary data at *Bioinformatics* online) and 1917 GOF SNPs (LOR < −1.77; Table 13, available as supplementary data at *Bioinformatics* online) that, respectively, altered the activity of 1623 ENCODE SCREEN enhancers (affecting 301 TFs) and 1247 enhancers between reference and alternative alleles, effectively converting enhancers to non-enhancers or vice versa. To check the clinical and regulatory importance of these variants, we compared them against the ClinVar, GWAS, and GTEx eQTL databases. We treated these overlaps as independent validation signals rather than definitive enrichment scores, as these catalogs are often biased toward protein-coding regions and leave many noncoding variants under-annotated. Even with these constraints, we found consistent functional evidence for several of our predicted variants. Of the identified LOF SNPs, eight possessed prior functional annotations, comprising three clinically significant variants and five significant eQTLs. Notably, all eight were predicted to disrupt binding sites for 25 different TFs (Tables 14 and 15, available as supplementary data at *Bioinformatics* online). Similarly, six GOF SNPs showed significant eQTL associations, supporting the role of enhancer gain in modulating gene expression.

Expanding the study to the areas immediately surrounding known SCREEN enhancers, we found 5464 “GOF” SNPs (Table 16, available as supplementary data at *Bioinformatics* online) predicted to flip nonfunctional DNA into active, enhancer-like states. These variants likely trigger 4057 brand-new enhancer activations. Notably, some of these SNPs are already linked to traits in GWAS and ClinVar databases or act as significant eQTLs (Table 17, available as supplementary data at *Bioinformatics* online), reinforcing the idea that mutations right next to enhancers can create entirely new regulatory signals.

**Figure 5:**
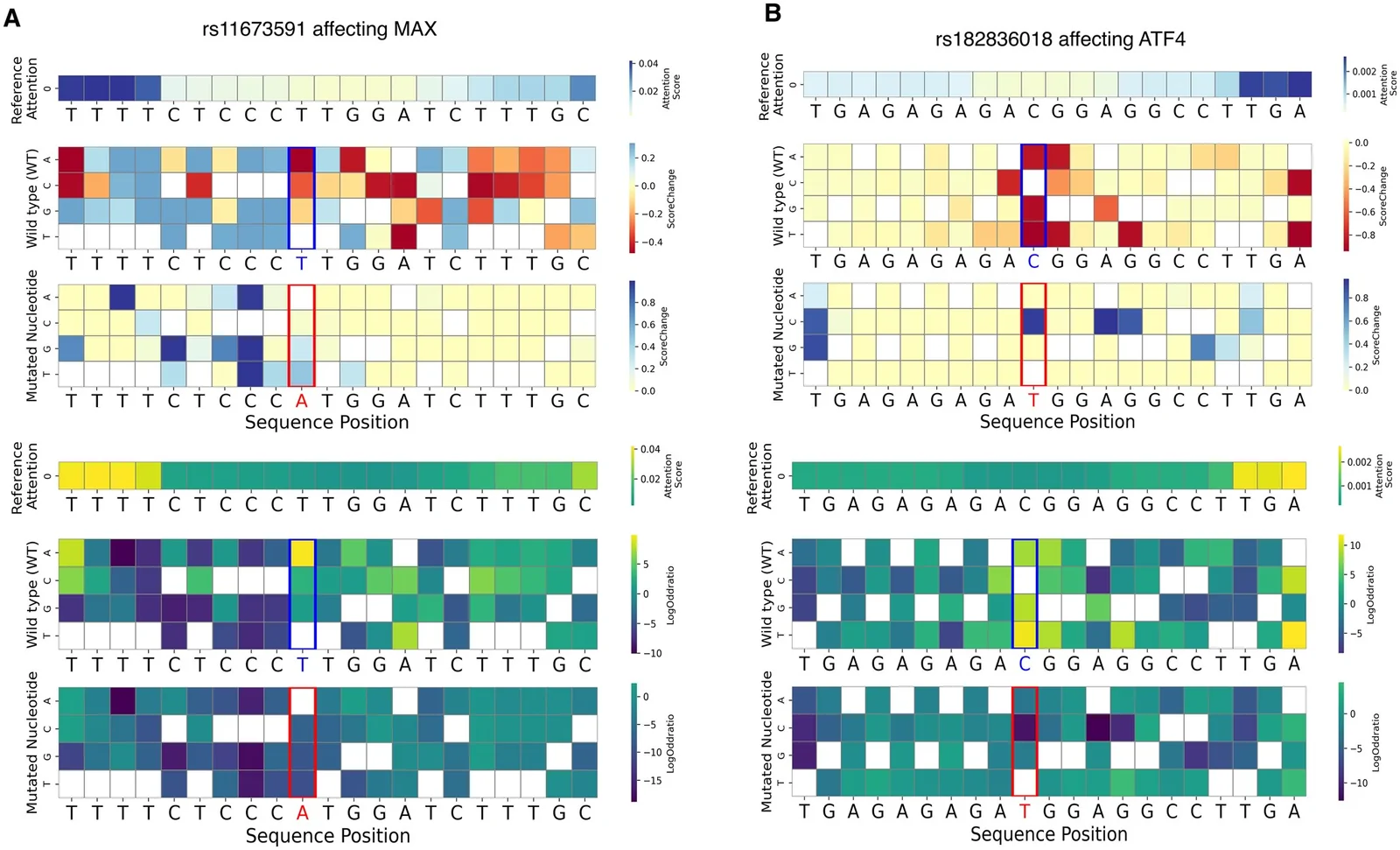
Effect of the functional genomic variants predicted by DNABERT-Enhancer on the TFBS within a given enhancer region. The mutation map depicts the score change (top 3) and the log odds ratio (bottom 3), for wild type and the mutation. (A) An intronic variant affecting MAX binding site targeting *PCAT19* gene. (B) An intronic variant in ATF4 binding site targeting *HS1BP3* gene.

Finally, we highlight two representative examples (Fig. 5). An intronic substitution variant mapped to *PCAT19* gene, reported as independent risk variant for prostate cancer in multi-ancestry or ancestry-specific analyses (Wang *et al.* 2023), was predicted to disrupt MAX (Myc-Associated Factor X) binding site. This variant was reported to influence the expression of *PCAT19* gene in multiple tissues including testis. Another intronic variant within an enhancer region was predicted to disrupt the ATF4 (activating TF 4) binding site, which was known to be associated with atrial fibrillation mapped to *HS1BP3* gene. In all the examples, DNABERT consistently shows highest attention at/around the variants of interest. The catalog of predicted variants presented here provide strong candidates for further experimental testing to confirm their functional impact on a specific trait or disease.

## 4 Discussion

Genome-wide prediction of enhancer regions and prioritization of genetic variants that affect the enhancer function are clinically significant and computationally challenging problems (Claringbould and Zaugg 2021). Publicly available databases, such as SCREEN, provide valuable resources for searching candidate regulatory elements, including enhancers, that were derived from ENCODE experimental data (The ENCODE Project Consortium *et al.* 2022). However, such experimentally curated enhancer data has limitations due to the finite number of genome-wide epigenetic experiments that can be performed in different cell types and states (Pennacchio *et al.* 2013). Most of the existing machine-learning based enhancer prediction models were trained on a small dataset, which was curated based on annotations of chromatin-state signatures in nine cell lines by ChromHMM (Ernst and Kellis 2012).

In this study, DNABERT-Enhancer showed improved performance across multiple evaluation settings. Notably, a model fine-tuned on a small subset (∼6%) of SCREEN enhancers recovered a substantial fraction of annotated enhancers genome wide. Fine-tuning large language models depends on high-quality labeled data; comparison of recent ENCODE cCRE versions shows many DHS regions previously unclassified are now annotated as enhancers, and this trend is likely to continue as more data emerge. Dataset composition, particularly the choice of negative sequences, strongly influenced performance. Consistent with prior work (Krutzfeldt *et al.* 2020), models trained with random genomic background as negatives performed best. Finally, models trained on longer sequences (350 bp) outperformed those trained on shorter ones (201 bp), suggesting longer enhancers capture greater regulatory complexity and sequence context (Li and Wunderlich 2017).

DNABERT-Enhancer consistently outperformed baseline and state-of-the-art methods by learning key enhancer features from large, curated datasets. Instead of relying on sequence similarity, its transformer-based attention mechanism captures complex motif combinations and long-range dependencies unique to enhancer regions. By training on aggregated SCREEN annotations, our global model enables genome-wide enhancer prediction without cell-type-specific inputs. This ensures broad applicability but sacrifices biological specificity by omitting contextual regulatory signals like chromatin accessibility, histone modifications, or TF binding. Therefore, predictions reflect sequence-based enhancer potential rather than cell-type–specific activity. To resolve this, we trained cell-type–specific models across four representative brain and breast lineages. Their similar performance indicates that key enhancer sequences are mostly conserved across cell types, capturing both shared and cell-specific regulatory signals. However, the scarcity of uniformly processed enhancer annotations across diverse cell lines severely limits the development of comprehensive, context-aware models. Our genome-wide predictions overlap significantly with public enhancer databases. However, because these databases often share the same experimental and annotation biases, the overlap does not fully prove context-independent generalizability across all tissues. Instead, these results validate that the model learned biologically relevant sequence features.

Beyond simply classifying genomic regions, DNABERT-Enhancer highlights high-attention sequences to pinpoint candidate variants that might disrupt enhancer function. Fusing these predictions with transcriptomic and chromatin interaction data offers a powerful strategy for mapping regulatory elements to cell-type-specific gene expression. This approach helps address a critical gap: while GWAS have uncovered millions of trait-associated variants, our ability to functionally interpret these noncoding variants remains severely limited (Yang *et al.* 2024). These findings emphasize the regulatory influence of noncoding variation, showing that even single-base changes can produce novel, active enhancers (Li *et al.* 2023). DNABERT-Enhancer predicts candidate SNPs that disrupt enhancer activity and TF binding (Section 3.8). These predictions help interpret noncoding variants of uncertain significance (VUSs), mitigating a major bottleneck in genomic medicine caused by scarce functional evidence for noncoding regions (Fowler and Rehm 2024). Recent benchmarking studies of variant effect predictors have further demonstrated that predictive performance is highly dependent on benchmark composition and remains substantially lower for noncoding regulatory variants than for coding variants, particularly when heterogeneous repositories such as dbSNP are used (Radjasandirane *et al.* 2024). Existing computational tools perform poorly across diverse noncoding variant classes. This highlights the need for enhancer-aware sequence models that can capture the regulatory grammar and contextual dependencies of enhancer function (Wang *et al.* 2022). Deep learning models offer a scalable method to prioritize candidate regulatory variants for experimental validation, addressing the vast amount of noncoding variation found in sequencing studies. Overlap with dbSNP, ClinVar, GWAS, and eQTL resources should be interpreted cautiously, as these datasets are biased toward well-characterized variants, overrepresentation of well-annotated genes and serve only as orthogonal support. The model may also generalize to rodent and non-human primate genomes without additional fine-tuning. Overall, this framework enables prioritization of potential causal variants for downstream validation and supports discovery of candidate enhancers for gene and cell therapies (Maeder and Gersbach 2016). In summary, DNABERT-Enhancer achieves strong performance in enhancer prediction and variant prioritization tasks, while highlighting both the potential and the current limitations of sequence-based models in capturing the complexity of gene regulation.

## Supplementary Material

btag456_Supplementary_Data

## Data Availability

The data and code underlying this article are available at https://github.com/DavuluriLab/DNABERT-Enhancer and have been archived and citable through https://zenodo.org/records/19157566. The trained model predictions can be explored interactively via the web application at https://dnabert-enhancer-datarepo.streamlit.app/.
