# Supplementary material for: Genomic language model for predicting enhancers and their allele-specific activity in the human genome
Source: Bioinformatics. 2026 Sep 10;42(9):btag456. doi: 10.1093/bioinformatics/btag456 (PMC13579158; doi:10.1093/bioinformatics/btag456)
Supplement: Supplementary file 1 [file btag456_supplementary_data.zip › Supplementary_data.docx]

**Supplementary information**

**Title:**

**Genomic language model for predicting enhancers and their allele-specific activity in the human genome**

Authors:

R. Sathian^1^, P. Dutta^1^, F. Ay^2,3,4^ and R.V. Davuluri^1, *^

Affiliations:

^1^ Department of Biomedical Informatics, Stony Brook University, Stony Brook, NY 11794, USA

^2^ Centers for Cancer Immunotherapy and Autoimmunity, La Jolla Institute for Immunology, La Jolla, CA 92037 USA

^3^ Bioinformatics and Systems Biology Graduate Program, University of California, San Diego, La Jolla, CA 92093 USA

^4^ Department of Pediatrics, University of California, San Diego, La Jolla, CA 92093 USA

**Supplementary Methods**

**Data sources:**

Two sets of datasets were utilized in this study. The enhancer region from the ENCODE Registry of Candidate *cis*-Regulatory Elements (cCREs) (V3), classified based on H3K27ac, H3K4me3, and CTCF signals across 25 fully classified and 839 partially classified human cell types. Second set is the enhancer sequences collected from both high-confidence experimental databases—DiseaseEnhancer(Zhang, et al., 2018), HACER (Wang, et al., 2019), RAEdb (Cai, et al., 2019), TiED (Xiong, et al., 2018), and VISTA (Visel, et al., 2007)—and predicted enhancer databases—EI cENHs (Pennacchio, et al., 2007), EnhancerAtlas(Gao and Qian, 2020), HEDD(Wang, et al., 2018), and SEdb (Zhang, et al., 2018). Sequences were derived from diverse experimental approaches, including CAGE, GRO-seq, PRO-seq, STARR-seq, and MPRA, or computational predictions. All sequences were converted to the GRCh38 human genome assembly using CrossMap (Zhao, et al., 2014) for hg19 and UCSC liftOver (Hinrichs, et al., 2006) for hg17. Redundant sequences with greater than 80% identity were excluded to ensure dataset uniqueness. Non-enhancer sequences (~68,750,000) were extracted from GRCh38 regions outside annotated enhancers, and both enhancer and non-enhancer sequences were fragmented to fixed lengths of 201 bp and 350 bp to generate separate datasets. To prevent model bias due to class imbalance, datasets were randomly down sampled to achieve a 1:1 ratio of enhancers to non-enhancers.

**Benchmarking Against Baseline classifiers**

To evaluate whether the transformer-based fine-tuning approach of DNABERT-Enhancer provides a meaningful advantage over conventional machine learning classifiers, we compared its performance against six widely used baseline methods: Random Forest (Breiman, 2001), *K*-Nearest Neighbor (KNN) (Mucherino, 2009), Gaussian Naive Bayes, AdaBoost Classifier, and Multi-layer Perceptron (MLP). All baseline classifiers were implemented using the scikit-learn library (Pedregosa, et al., 2011). For the KNN classifier, the number of neighbors (k) was optimized via 3-fold stratified cross-validation on the training set using RandomizedSearchCV with ROC-AUC as the scoring criterion, evaluating k ∈ {3, 5, 7, 9}. The optimal k was 5 for ESd-201 (uniform weighting, Euclidean distance) and 3 for ESd-350 (distance weighting, Manhattan distance). All other baseline classifiers were similarly tuned using 3-fold stratified cross-validation with focused hyperparameter ranges.

To ensure a fair comparison, we used DNABERT embeddings as input features for all baseline classifiers, since these methods cannot directly process raw DNA sequences. Specifically, each DNA sequence in the training and test sets was passed through the pre-trained DNABERT model, and the [CLS] token representation (a 768-dimensional vector) from the final hidden layer was extracted as the input feature vector. This design isolates the contribution of the classification algorithm from the sequence representation, allowing a direct assessment of whether the end-to-end fine-tuning approach of DNABERT-Enhancer offers an advantage over attaching conventional classifiers to the same learned representations. Both the ESd-201 and ESd-350 datasets were used for training and evaluation, with the same train-test split applied to DNABERT-Enhancer. Model performance was assessed using accuracy, precision, recall, F1 score, and Matthews correlation coefficient (MCC).

**Benchmarking Against Published Enhancer Prediction Methods**

To assess the performance of DNABERT-Enhancer relative to existing methods, we compared our model with three recently published enhancer prediction frameworks: iEnhancer-ELM (Li, et al., 2023), iEnhancer-DCLA (Liao, et al., 2022), and iEnhancer-ECNN (Nguyen, et al., 2019). All three methods were originally trained on a benchmark dataset containing 1,484 enhancers and 1,484 non-enhancers constructed based on chromatin state annotations derived from ChromHMM (Ernst and Kellis, 2012). Our pre-trained DNABERT model was also employed by iEnhancer-ELM model, but a 2-layer perceptron network was attached to the model for classification. Like iEnhancer-ELM, iEnhancer-ECNN is an ensemble method, but the later uses CNN model architecture. iEnhancer-DCLA also utilizes CNN along with Bi-LSTM and attention mechanism to extract features for classification.

To ensure an unbiased comparison, we evaluated the published models on both ESd-201-Test and ESd-350-Test datasets. Where available, publicly released source codes or reported model configurations were used. To minimize bias arising from sequence length differences, both 201 bp and 350 bp test datasets were used for evaluation. All models were assessed using identical performance metrics and evaluation protocols.

**Benchmarking Against Nucleotide transformer**

To benchmark DNABERT-Enhancer against a recent large-scale genomic foundation model, we compared its performance with the Nucleotide Transformer (NT) (Dalla-Torre, et al., 2025), a family of transformer-based models pre-trained on DNA sequences with model sizes ranging from 50 million to 2.5 billion parameters. We selected the NT 500M Human Reference model (*InstaDeepAI/nucleotide-transformer-500m-human-ref*), which was pre-trained on the human reference genome, for comparison.

The NT 500M model was fine-tuned on our benchmark enhancer datasets (ESd-201-Train and ESd-350-Train) using a protocol consistent with the one applied for DNABERT-Enhancer. Input sequences were tokenized using the NT tokenizer, which employs a non-overlapping 6-mer tokenization scheme with a vocabulary of 4,096 tokens. A classification head consisting of a linear projection followed by softmax activation was attached to the model’s output for binary enhancer/non-enhancer classification. To identify optimal hyperparameters, we performed a Bayesian hyperparameter search using Optuna over 4 trials, exploring learning rates (1×10⁻⁵ to 1×10⁻³), number of training epochs (8–20), weight decay (1×10⁻⁶ to 1×10⁻²), and warmup fraction (5–10% of total training steps). The best-performing configuration used a learning rate of 5×10⁻⁴, weight decay of 2.14×10⁻³, 17 training epochs, and 3,688 warmup steps. Training was performed with a per-device batch size of 30 across 8 NVIDIA A40 GPUs (effective batch size of 240), using mixed-precision (FP16) training with the AdamW optimizer. The best model was selected based on validation loss, and performance was evaluated on the held-out test set (ESd-350-Test) using accuracy, precision, recall, F1 score, and MCC.

**Application of DNABERT-enhancer model on the human**

To validate the efficiency of the model in genome-wide analysis, we downloaded the human genome sequence data (GRCh38) from UCSC Genome Browser. Each chromosome was processed using a sliding window approach, with a window size of 350 bp and a shift of 50 bp (~86% overlap). This configuration improves prediction resolution while maintaining robustness through overlapping sequence context. Sequences containing ‘N’ nucleotides were dropped from the data as they represent unidentified bases. The data was then tokenized to hexamers (6-mer), followed by random labeling, and were processed using the best fine-tuned enhancer model for the prediction. Once the prediction results were generated for subsequences $\left( S_{i}|i\in\left[ 1,2,3\ldots m \right] \right)$, where $m$ is the total number of subsequences, we deduced the probability score $P\left( W_{j} \right)$ for every 50bp window $\left( W_{j}|j\in\left[ 1,2,3\ldots n \right] \right)$, where $n$ is the total number of windows. The window was labeled as an enhancer if its probability $P\left( W_{j} \right)\geq0.5$. When adjacent windows also satisfied the threshold, they were merged to extend the predicted region. If a window with probability $P\left( W_{j} \right)<0.5$ was encountered, subsequent windows were examined to determine whether this represented a true boundary or a brief fluctuation in prediction scores. A region was terminated only when a sustained stretch of consecutive low-probability windows, approximately spanning the enhancer window length based on the sliding step size, was observed; otherwise, short dips in probability were treated as noise and the windows were merged into the ongoing enhancer region. The predicted enhancer regions were then evaluated by overlapping with the enhancer data cataloged in the open-source databases, at different overlap cutoffs (in percentage; 5,10,15,20,25,50,75,95 and All). Significance of the observed overlaps were assessed through permutation testing (Ernst, 2004). Let $O_{obs}$ denote the observed overlap (in base pairs) between predicted enhancers and a given database. To generate the null distribution, we randomly sampled regions from the genome 1,000 times, preserving both chromosome-wise distributions and the original region lengths of the predicted enhancers, while excluding ENCODE blacklist regions(Amemiya, Kundaje and Boyle, 2019). Let $O_{j}^{null}$ denote the overlap from the permutation ( $j=1,2\ldots N_{perm}$, where $N_{perm}$ is the total number of permutations). All genomic interval operations, including the generation of random regions and overlap calculations, were implemented in Python using the pybedtools library(Dale, Pedersen and Quinlan, 2011). Statistical computations such as permutation testing and p-value estimation were performed with NumPy (Harris, et al., 2020) and SciPy(Virtanen, et al., 2020). The observed overlap was then compared against this null distribution to calculate fold enrichment:

$$Fold enrichment=\frac{O_{obs}}{mean(O_{j}^{null})}$$

and the empirical p-values:

$$P_{emperical}=\frac{1+\sum_{j=1}^{N_{perm}} 1\left\{ O_{j}^{null}\geq O_{obs} \right\}}{N_{perm}+1}$$

The minimum attainable nonzero p-value with 1,000 permutations was 0.001 (reported as 0.00099).

**Variant Effect Prediction**

To predict the effect of genetic variants within the enhancers and the TF target regions, we downloaded the dbSNP release 155(GRCh38) (Sherry, Ward and Sirotkin, 1999) and extracted short variants located in these regions. For enhancer regions, prediction probabilities were recomputed by substituting the variant nucleotide into the sequence and evaluating with DNABERT-Enhancer-350. For TF target regions within enhancers, we followed the same procedure using DeepVRegulome(Dutta, et al., 2024), a deep-learning framework for functional variant interpretation in the human regulome. Briefly, the DeepVRegulome includes 700 DNABERT finetuned models trained on 4,228 ChIP-seq experiments of 667 transcription factors and 33 histone marks obtained from The Encyclopedia of DNA Elements (ENCODE) database(The ENCODE Project Consortium, 2012). To identify gain-of-function (GOF) regulatory variants in non-enhancer regions, we used the flanking sequences of SCREEN enhancers as an appropriate background. Variants in these flanking regions were evaluated with DNABERT-Enhancer-350 to detect GOF effects that increased enhancer-like predictions relative to the reference sequence, providing a complementary perspective on regulatory variation outside canonical enhancer loci.

To classify functional variants, we initially applied a probability threshold of 0.5 between reference and alternate alleles. We further benchmarked variant effects by calculating score changes and log-odds ratios (LOR). The variant effect scores were defined following the original paper, DNABERT(Ji, et al., 2021). Briefly, was calculated as the normalized probability difference:

$$s= \Delta p= \frac{p\left( s^{'} \right)- p\left( s \right)}{max\left( p\left( s^{'} \right),p\left( s \right) \right)}$$

where $p\left( s \right)$ is the prediction of sequence, $p\left( s^{'} \right)$is the prediction of substituted sequence and the max term is added to amplify the strong effects of certain genetic variants. The Log odds ratio quantified the relative change in probability as:

$$OR =log2\frac{p\left( s \right)}{1-p\left( s \right)} -log2\frac{p\left( s^{'} \right)}{1-p\left( s^{'} \right)}$$

Where LOR $=$ 0 indicates no association between the events. These scores serve as a measure to further filter high confidence among the candidate variants. To formally quantify significance, let $T_{i}$ denotes LOR of variant $i$, for $i=1,2\ldots N$, where $N$ is the total number of variants. The thresholds were defined as

$$x_{1}= \mu+k\sigma for Loss of function \left( \mathrm{LOF} \right), x_{2}= \mu-k\sigma for Gain of function \left( \mathrm{GOF} \right)$$

where $\mu$ and $\sigma$ denote the mean and standard deviation of the LOR distribution, respectively, and $k$ represents the number of standard deviations ($k=1,2 .. 5$). The number of variants exceeding these thresholds was calculated using the indicator function:

$$N_{thr}^{LOF}=\sum_{i=1}^{N} 1\left\{ T_{i}\geq x_{1} \right\}, N_{thr}^{GOF}=\sum_{i=1}^{N} 1\left\{ T_{i}\leq x_{2} \right\}$$

where, $N_{thr}$ is the number of variants which exceeds the LOR threshold. The empirical p-value for each observed LOR was then estimated as

$$P_{emperical}^{LOF}=\frac{N_{thr}^{LOF}}{N} , P_{emperical}^{GOF}=\frac{N_{thr}^{GOF}}{N}$$

providing a formal measure of statistical significance and allowing us to identify variants with the strongest predicted regulatory impact. All sequence manipulations, probability calculations, and statistical analyses were performed in Python using NumPy (Harris, et al., 2020) and SciPy (Virtanen, et al., 2020) for numerical computation.

To connect candidate variants with known functional and clinical annotation, we queried the dbSNP_clinvar155 track, ClinVar (Landrum, et al., 2018) and GWAS catalogue (Uffelmann, Posthuma and Peyrot, 2023). In addition, we assessed the expression quantitative trait loci (eQTL) using the UCSC tracks for high-confidence cis-eQTLs from 49 GTEx tissues (V8 data release). Specifically, we leveraged CAVIAR confidence set (consist of 526,252 unique high causal variants with causal posterior probability $>$ 0.1), and the DAP-G track (consists of 1,451,916 unique variants with strong eQTLs signals, signal-level posterior inclusion probability (SPIP) $>$ 0.95), totaling to 1,674,664 variants.

**Evaluation metrics**

The performance of all the models was measured by 5 performance metrics: $acc=\frac{TP+TN}{TP+TN+FP+FN}$ , $precision=\frac{TP}{TP+FN}$, $recall=\frac{TP}{TP+FP}$, $mcc=\frac{TN\times TP-FN\times FP}{\sqrt{\left( TP+FP \right)\left( TP+FN \right)\left( TN+FP \right)\left( TN+FN \right)}}$, $F1= 2\times\frac{Precision\times Recall}{Precision+Recall}$*,* where TP is true positive, TN is true negative, FP is false positive, and FN is the false negative values.

**Supplementary Results**

**Effect of classification threshold on model performance:**

To evaluate the impact of classification threshold on model performance, we computed accuracy, precision, recall, F1-score, and Matthews Correlation Coefficient (MCC) across a range of thresholds (0.1–0.9) (**Supplementary Fig. S2A**). As expected, increasing the threshold resulted in higher precision but lower recall, reflecting the trade-off between false positives and false negatives. Composite metrics such as F1-score and MCC, which balance sensitivity and precision, reached their maximum values at a threshold of approximately 0.4, with minimal differences observed at 0.5. This indicates that the range of ~0.4–0.5 provides an optimal balance of performance. The commonly used default threshold of 0.5 lies within this range and yields near-maximal performance, supporting its use in our analysis.

**Evaluation of Model Robustness Using Alternative Background Sequences:**

To evaluate the robustness of our model and assess the potential influence of background sequence selection on model performance, we trained additional DNABERT-Enhancer-350 models with alternative negative sets. In the first alternate version, the non-enhancer regions were sampled from random genomic locations excluding only the high-confidence enhancer regions, while the positive set of 350 bp enhancer sequences remained unchanged. This refined model, named DNABERT-Enhancer-350_refined achieved a comparable accuracy of 87.8%, closely matching the original model’s performance (**Figure 2D**), suggesting that the original model is not heavily biased by weak regulatory features in the background and is likely learning enhancer-specific sequence characteristics. In a second version, we restricted the negative set to DNase I hypersensitive (DHS) or open chromatin regions that do not overlap enhancers in any tissue or cell type. This model, named DNABERT-Enhancer-350_dhs achieved a lower accuracy of 61.5% (**Figure 2D**), reflecting the greater difficulty of distinguishing enhancers from other accessible but non-enhancer regions. We note, however, that the cCRE classification in ENCODE SCREEN has been updated over time as more experimental data and cell types are incorporated. For example, 11,636 DHSs that were classified as non-enhancers in SCREEN v3 (2022) are now annotated as enhancers in SCREEN v4 (2025). Notably, among the ~11,000 DHSs, 279 regions of 350 bp length were evaluated, and our model predicted 86.37% of them as enhancers. This suggests that some DHSs previously labeled as non-enhancers may in fact represent true enhancers that were not yet annotated. Together, these results demonstrate that our predictive framework trained on ENCODE cCRE enhancers, with random genomic sequences as non-enhancer regions, is most accurate for genome-wide prediction of enhancers.

**Permutation-based validation of enhancer overlap enrichment:**

To evaluate whether the overlap between predicted enhancer regions and enhancers in the public databases could be due to chance, we performed permutation testing (details in supplementary methods). The testing was done by randomly selecting genomic regions (N=1000) to calculate the fold enrichment and empirical p-value. Across all databases tested, the observed overlaps showed significant enrichment (fold enrichment ranging from 1.35 to 2.80; empirical p-value ≤ 0.001 (N=1000 permutations); **Supplementary Table S6**) compared to the null expectation, thereby confirming that the concordance between our predictions and external datasets reflects genuine biological relevance rather than broad genomic coverage.

**References**

Amemiya, H.M., Kundaje, A. and Boyle, A.P. The ENCODE Blacklist: Identification of Problematic Regions of the Genome. *Sci Rep* 2019;9(1):9354.

Breiman, L. Random forests. *Mach Learn* 2001;45(1):5-32.

Cai, Z.*, et al.* RAEdb: a database of enhancers identified by high-throughput reporter assays. *Database (Oxford)* 2019;2019.

Consortium, E.P. An integrated encyclopedia of DNA elements in the human genome. *Nature* 2012;489(7414):57-74.

Dale, R.K., Pedersen, B.S. and Quinlan, A.R. Pybedtools: a flexible Python library for manipulating genomic datasets and annotations. *Bioinformatics* 2011;27(24):3423-3424.

Dalla-Torre, H.*, et al.* Nucleotide Transformer: building and evaluating robust foundation models for human genomics. *Nat Methods* 2025;22(2):287-297.

Dutta, P.*, et al.* Leveraging genomic foundation model to predict small somatic mutations that impact non-coding functional sites in cancer genomes. In.; 2024.

Ernst, J. and Kellis, M. ChromHMM: automating chromatin-state discovery and characterization. *Nat Methods* 2012;9(3):215-216.

Ernst, M.D. Permutation methods: A basis for exact inference. *Stat Sci* 2004;19(4):676-685.

Gao, T. and Qian, J. EnhancerAtlas 2.0: an updated resource with enhancer annotation in 586 tissue/cell types across nine species. *Nucleic Acids Res* 2020;48(D1):D58-D64.

Harris, C.R.*, et al.* Array programming with NumPy. *Nature* 2020;585(7825):357-362.

Hinrichs, A.S.*, et al.* The UCSC Genome Browser Database: update 2006. *Nucleic Acids Res* 2006;34(Database issue):D590-598.

Ji, Y., Zhou, Z., Liu, H. and Davuluri, R.V. DNABERT: pre-trained Bidirectional Encoder Representations from Transformers model for DNA-language in genome. *Bioinformatics* 2021;37(15):2112-2120.

Landrum, M.J.*, et al.* ClinVar: improving access to variant interpretations and supporting evidence. *Nucleic Acids Res* 2018;46(D1):D1062-D1067.

Li, J.*, et al.* iEnhancer-ELM: improve enhancer identification by extracting position-related multiscale contextual information based on enhancer language models. *Bioinform Adv* 2023;3(1):vbad043.

Liao, M., Zhao, J.P., Tian, J. and Zheng, C.H. iEnhancer-DCLA: using the original sequence to identify enhancers and their strength based on a deep learning framework. *BMC Bioinformatics* 2022;23(1):480.

Mucherino, A. k-Nearest Neighbor Classification. *Springer Ser Optim A* 2009;34:83-106.

Nguyen, Q.H.*, et al.* iEnhancer-ECNN: identifying enhancers and their strength using ensembles of convolutional neural networks. *BMC Genomics* 2019;20(Suppl 9):951.

Pedregosa, F.*, et al.* Scikit-learn: Machine Learning in P ython. *Journal of Machine Learning Research* 2011;12:2825--2830.

Pennacchio, L.A., Loots, G.G., Nobrega, M.A. and Ovcharenko, I. Predicting tissue-specific enhancers in the human genome. *Genome Res* 2007;17(2):201-211.

Sherry, S.T., Ward, M. and Sirotkin, K. dbSNP-database for single nucleotide polymorphisms and other classes of minor genetic variation. *Genome Res* 1999;9(8):677-679.

Uffelmann, E., Posthuma, D. and Peyrot, W.J. Genome-wide association studies of polygenic risk score-derived phenotypes may lead to inflated false positive rates. *Sci Rep* 2023;13(1):4219.

Virtanen, P.*, et al.* SciPy 1.0: fundamental algorithms for scientific computing in Python. *Nat Methods* 2020;17(3):261-272.

Visel, A., Minovitsky, S., Dubchak, I. and Pennacchio, L.A. VISTA Enhancer Browser--a database of tissue-specific human enhancers. *Nucleic Acids Res* 2007;35(Database issue):D88-92.

Wang, J.*, et al.* HACER: an atlas of human active enhancers to interpret regulatory variants. *Nucleic Acids Res* 2019;47(D1):D106-D112.

Wang, Z.*, et al.* HEDD: Human Enhancer Disease Database. *Nucleic Acids Res* 2018;46(D1):D113-D120.

Xiong, L.*, et al.* Genome-wide Identification and Characterization of Enhancers Across 10 Human Tissues. *Int J Biol Sci* 2018;14(10):1321-1332.

Zhang, G.*, et al.* DiseaseEnhancer: a resource of human disease-associated enhancer catalog. *Nucleic Acids Res* 2018;46(D1):D78-D84.

Zhao, H.*, et al.* CrossMap: a versatile tool for coordinate conversion between genome assemblies. *Bioinformatics* 2014;30(7):1006-1007.

**Supplementary Figures**

**Supplementary** **Fig S1:** Distribution of distances from transcription start sites for positive (Enhancers) and negative (Non-enhancers) sequences in the (A) ESd-201 and (B) ESd-350. Although negative sequences are mostly enriched at larger distances from TSS, the central mass of both distribution lies between 1-100 kb, indicating the model performance is not trivially driven by promoter proximity.

**Supplementary** **Fig S2:** (A) Performance metrics (accuracy, precision, recall, F1-score, and MCC) evaluated across a range of classification thresholds (0.1–0.9) (B) Loss Progression of our best DNABERT-Enhancer models: DNABERT-Enhancer-201 (left) and DNABERT-Enhancer-350 (right), indicating a trend of improvement. As the number of steps increases, the model effectively reduces the loss. A comprehensive overview on the progression of the performance of our best DNABERT-Enhancer models (C) DNABERT-Enhancer-201 trained on ESd-201-Train (D) DNABERT-Enhancer-350 trained on ESd-350-Train data over training steps, in terms of performance metrics namely, Accuracy, Precision, Recall, F1 score and MCC.

**Supplementary Figure S3:** (A) Length distribution of the enhancers in the SCREEN data which are not mapped to the predicted enhancers projecting ~98% of data has length less than 350 bp. (B) Heatmap of the attention regions learned by DNABERT-Enhancer-350 showing the absence of any positional preference within enhancer regions (C) Upset plot showing the intersection of predicted enhancer regions (which does not map to SCREEN data as part of validation) with different publicly available datasets.

**Supplementary tables**

**Table S1**. Hyperparameters used for the fine-tuning of base-line classifiers on ESd-201 and ESd-350 dataset

| Method | Parameter | ESd-201 | ESd-350 |
| --- | --- | --- | --- |
| Random Forest | n_estimators | 200 | 300 |
|  | max_depth | 25 | 25 |
|  | min_samples_split | 2 | 2 |
| kNN | n_neighbors | 5 | 3 |
|  | weights | uniform | distance |
|  | metric | euclidean | manhattan |
| AdaBoost | n_estimators | 100 | 200 |
|  | learning_rate | 0.1 | 0.5 |
| Gaussian NB | (no tuning needed) | - | - |
| Multi-layer Perceptron | hidden | (128,64) | (256,128) |
|  | lr | 0.001 | 0.01 |
|  | alpha | 0.01 | 0.01 |
|  | batch | 64 | 64 |

**Table S2**. Comparison of DNABERT-Enhancer-201 and DNABERT-Enhancer-350 to the state-of-the-art methods namely, iEnhancer-ECNN, iEnhancer-DCLA and iEnhancer-ELM

| Models | Model performance on ESd-201-Test | | | | | | Model performance on ESd-350-Test | | | | | |
| --- | --- | --- | --- | --- | --- | --- | --- | --- | --- | --- | --- | --- |
|  | **Accuracy (%)** | **Precision (%)** | **Specificity (%)** | **Recall (%)** | **F1 score (%)** | **mcc (%)** | **Accuracy (%)** | **Precision (%)** | **Specificity (%)** | **Recall (%)** | **F1 score (%)** | **mcc (%)** |
| iEnhancer-ECNN | 70.58 | 64.59 | 76.56 | 73.37 | 68.7 | 41.45 | 72.92 | 69.24 | 76.6 | 74.74 | 71.88 | 45.97 |
| iEnhancer-DCLA | 67.41 | 55.79 | 79.04 | 72.69 | 63.13 | 35.8 | 71.23 | 60.6 | 81.81 | 76.93 | 67.82 | 37.5 |
| iEnhancer-ELM | 71.24 | 51.62 | 90.86 | 84.95 | 64.22 | 46.18 | 79.08 | 72.04 | 86.11 | 83.84 | 77.49 | 58.74 |
| DNABERT-Enhancer | 82.04 | 78.29 | 85.7 | 84.64 | 81.3 | 64.27 | 88.05 | 85.29 | 90.81 | 90.27 | 87.71 | 76.22 |

**Table S3.** Whole genome prediction: The chromosome wise statistics of enhancer elements predicted by DNABERT-Enhancer

| Chromosome | #Total subsequences generated for prediction | #Total subsequences predicted as enhancer | #Total predicted enhancer regions | Predicted enhancer regions in base pair (bp) |
| --- | --- | --- | --- | --- |
| chr1 | 4,611,410 | 1,180,468 | 134,180 | 70,247,700 |
| chr2 | 4,811,834 | 1,121,966 | 142,013 | 67,267,150 |
| chr3 | 3,999,029 | 833,928 | 115,411 | 50,417,900 |
| chr4 | 3,814,233 | 656,520 | 104,420 | 39,350,000 |
| chr5 | 3,627,072 | 748,122 | 103,248 | 45,034,100 |
| chr6 | 3,419,721 | 719,433 | 100,205 | 43,293,750 |
| chr7 | 3,265,665 | 725,612 | 92,640 | 43,088,200 |
| chr8 | 2,929,297 | 661,426 | 85,831 | 39,627,550 |
| chr9 | 2,442,596 | 610,609 | 70,350 | 36,082,550 |
| chr10 | 2,669,791 | 713,054 | 78,831 | 42,558,000 |
| chr11 | 2,702,080 | 684,957 | 74,476 | 40,627,600 |
| chr12 | 2,671,678 | 602,246 | 77,220 | 35,937,950 |
| chr13 | 1,959,739 | 377,823 | 54,866 | 22,732,600 |
| chr14 | 1,811,410 | 423,962 | 51,110 | 25,260,400 |
| chr15 | 1,741,356 | 459,569 | 50,043 | 27,480,600 |
| chr16 | 1,687,790 | 534,232 | 49,974 | 31,131,600 |
| chr17 | 1,770,537 | 539,536 | 46,912 | 31,095,300 |
| chr18 | 1,691,288 | 347,022 | 45,683 | 20,929,900 |
| chr19 | 1,169,060 | 395,954 | 33,457 | 22,355,900 |
| chr20 | 1,306,365 | 412,544 | 37,812 | 24,391,450 |
| chr21 | 803,100 | 198,592 | 21,660 | 11,619,750 |
| chr22 | 783,280 | 302,543 | 20,546 | 17,289,350 |
| chrX | 3,161,699 | 535,082 | 79,833 | 32,421,900 |
| chrY | 531,238 | 95,081 | 13,874 | 5,787,900 |
| **Total** | **59,381,268** | **13,880,281** | **1,684,595** | **826,029,100 bp**  **(26.65% of Human genome)** |

**Table S4.** Whole genome prediction results: intersection of predicted enhancer regions with the enhancer regions in ENCODE cCREs (#961,227) available through SCREEN

| Prediction probability | #Total unique predicted enhancers | #SCREEN enhancers intersected with the prediction | SCREEN intersect (%) | #Predicted enhancers intersected with the SCREEN | prediction intersect (%) | Remaining SCREEN Enhancers |
| --- | --- | --- | --- | --- | --- | --- |
| >=0.5 | 1,684,595 | 788,686 | 82.05 | 428,489 | 25.44 | 172,541 |
| >=0.55 | 1,648,417 | 772,290 | 80.34 | 426,711 | 25.89 | 188,937 |
| >=0.6 | 1,606,418 | 754,524 | 78.50 | 423,921 | 26.39 | 206,703 |
| >=0.65 | 1,556,996 | 734,608 | 76.42 | 419,691 | 26.96 | 226,619 |
| >=0.7 | 1,498,955 | 712,427 | 74.12 | 413,980 | 27.62 | 248,800 |
| >=0.75 | 1,429,888 | 685,931 | 71.36 | 406,109 | 28.40 | 275,296 |
| >=0.8 | 1,343,371 | 653,937 | 68.03 | 395,405 | 29.43 | 307,290 |
| >=0.85 | 1,232,286 | 612,692 | 63.74 | 379,434 | 30.79 | 348,535 |
| >=0.9 | 1,075,849 | 552,320 | 57.46 | 352,874 | 32.80 | 408,907 |

**Table S5**. Whole genome prediction results: intersection of predicted enhancer regions (#1,684,595) with the enhancer regions reported in high confidence databases

| Database | #Total enhancers in database (All chromosome) | Prediction probability | #Enhancers in database intersected with the prediction | Database intersect (%) | #Predicted enhancers intersected with the database | Prediction intersect (%) |
| --- | --- | --- | --- | --- | --- | --- |
| DiseaseEnhancer | 497 | >=0.5 | 447 | 89.94 | 1,391 | 0.08 |
|  |  | >=0.55 | 445 | 89.54 | 1,413 | 0.09 |
|  |  | >=0.6 | 443 | 89.13 | 1,417 | 0.09 |
|  |  | >=0.65 | 438 | 88.13 | 1,411 | 0.09 |
|  |  | >=0.7 | 431 | 86.72 | 1,425 | 0.1 |
|  |  | >=0.75 | 425 | 85.51 | 1,403 | 0.1 |
|  |  | >=0.8 | 413 | 83.1 | 1,349 | 0.1 |
|  |  | >=0.85 | 404 | 81.29 | 1,325 | 0.11 |
|  |  | >=0.9 | 379 | 76.26 | 1,242 | 0.12 |
| HACER | 224,444 | >=0.5 | 170,554 | 75.99 | 233,685 | 13.87 |
|  |  | >=0.55 | 168,022 | 74.86 | 229,559 | 13.93 |
|  |  | >=0.6 | 165,420 | 73.7 | 224,780 | 13.99 |
|  |  | >=0.65 | 162,563 | 72.43 | 218,864 | 14.06 |
|  |  | >=0.7 | 159,463 | 71.05 | 212,094 | 14.15 |
|  |  | >=0.75 | 156,023 | 69.52 | 203,810 | 14.25 |
|  |  | >=0.8 | 151,993 | 67.72 | 193,617 | 14.41 |
|  |  | >=0.85 | 146,628 | 65.33 | 179,910 | 14.6 |
|  |  | >=0.9 | 138,857 | 61.87 | 160,112 | 14.88 |
| RAEdb | 297,523 | >=0.5 | 225,199 | 75.69 | 29,223 | 1.73 |
|  |  | >=0.55 | 220,069 | 73.97 | 28,811 | 1.75 |
|  |  | >=0.6 | 214,779 | 72.19 | 28,378 | 1.77 |
|  |  | >=0.65 | 208,557 | 70.1 | 27,872 | 1.79 |
|  |  | >=0.7 | 202,044 | 67.91 | 27,291 | 1.82 |
|  |  | >=0.75 | 193,915 | 65.18 | 26,578 | 1.86 |
|  |  | >=0.8 | 184,574 | 62.04 | 25,690 | 1.91 |
|  |  | >=0.85 | 172,458 | 57.96 | 24,403 | 1.98 |
|  |  | >=0.9 | 155,532 | 52.28 | 22,554 | 2.1 |
| TiED | 53,331 | >=0.5 | 46,345 | 86.9 | 37,400 | 2.22 |
|  |  | >=0.55 | 45,654 | 85.6 | 37,379 | 2.27 |
|  |  | >=0.6 | 44,901 | 84.19 | 37,289 | 2.32 |
|  |  | >=0.65 | 44,032 | 82.56 | 37,061 | 2.38 |
|  |  | >=0.7 | 43,049 | 80.72 | 36,842 | 2.46 |
|  |  | >=0.75 | 41,878 | 78.52 | 36,478 | 2.55 |
|  |  | >=0.8 | 40,463 | 75.87 | 35,897 | 2.67 |
|  |  | >=0.85 | 38,580 | 72.34 | 34,964 | 2.84 |
|  |  | >=0.9 | 35,720 | 66.98 | 33,395 | 3.1 |
| Vista | 941 | >=0.5 | 930 | 98.83 | 1,626 | 0.1 |
|  |  | >=0.55 | 929 | 98.72 | 1,634 | 0.1 |
|  |  | >=0.6 | 926 | 98.41 | 1,631 | 0.1 |
|  |  | >=0.65 | 921 | 97.87 | 1,619 | 0.1 |
|  |  | >=0.7 | 916 | 97.34 | 1,614 | 0.11 |
|  |  | >=0.75 | 906 | 96.28 | 1,600 | 0.11 |
|  |  | >=0.8 | 900 | 95.64 | 1,577 | 0.12 |
|  |  | >=0.85 | 881 | 93.62 | 1,551 | 0.13 |
|  |  | >=0.9 | 850 | 90.33 | 1,470 | 0.14 |

**Table S6**. Whole genome prediction results: intersection of predicted enhancer regions (#1,684,595) with the predicted enhancer regions reported in databases

| Database | #Total enhancers in database (All chromosome) | Prediction probability | #Enhancers in database intersected with the prediction | Database intersect (%) | #Predicted enhancers intersected with the database | Prediction intersect (%) |
| --- | --- | --- | --- | --- | --- | --- |
| EI | 4,404 | >=0.5 | 3,768 | 85.56 | 3,891 | 0.23 |
|  |  | >=0.55 | 3,702 | 84.06 | 3,828 | 0.23 |
|  |  | >=0.6 | 3,623 | 82.27 | 3,760 | 0.23 |
|  |  | >=0.65 | 3,551 | 80.63 | 3,690 | 0.24 |
|  |  | >=0.7 | 3,453 | 78.41 | 3,598 | 0.24 |
|  |  | >=0.75 | 3,346 | 75.98 | 3,462 | 0.24 |
|  |  | >=0.8 | 3,203 | 72.73 | 3,310 | 0.25 |
|  |  | >=0.85 | 3,016 | 68.48 | 3,103 | 0.25 |
|  |  | >=0.9 | 2,745 | 62.33 | 2,806 | 0.26 |
| EnhancerAtlas | 153,507 | >=0.5 | 117,684 | 76.66 | 926,175 | 54.98 |
|  |  | >=0.55 | 115,722 | 75.39 | 902,131 | 54.73 |
|  |  | >=0.6 | 113,526 | 73.95 | 874,779 | 54.46 |
|  |  | >=0.65 | 111,128 | 72.39 | 843,622 | 54.18 |
|  |  | >=0.7 | 108,328 | 70.57 | 807,477 | 53.87 |
|  |  | >=0.75 | 105,158 | 68.5 | 764,828 | 53.49 |
|  |  | >=0.8 | 101,314 | 66 | 712,439 | 53.03 |
|  |  | >=0.85 | 96,198 | 62.67 | 646,358 | 52.45 |
|  |  | >=0.9 | 88,751 | 57.82 | 555,237 | 51.61 |
| HEDD | 2,568,533 | >=0.5 | 1,622,862 | 63.18 | 671,476 | 39.86 |
|  |  | >=0.55 | 1,564,552 | 60.91 | 664,561 | 40.32 |
|  |  | >=0.6 | 1,503,706 | 58.54 | 655,500 | 40.81 |
|  |  | >=0.65 | 1,438,577 | 56.01 | 643,592 | 41.34 |
|  |  | >=0.7 | 1,368,374 | 53.27 | 628,629 | 41.94 |
|  |  | >=0.75 | 1,289,529 | 50.2 | 609,720 | 42.64 |
|  |  | >=0.8 | 1,198,071 | 46.64 | 584,839 | 43.54 |
|  |  | >=0.85 | 1,086,301 | 42.29 | 549,960 | 44.63 |
|  |  | >=0.9 | 938,172 | 36.53 | 496,608 | 46.16 |
| SEdb_TE | 17,141,114 | >=0.5 | 15,573,721 | 90.86 | 1,293,138 | 76.76 |
|  |  | >=0.55 | 15,437,110 | 90.06 | 1,269,820 | 77.03 |
|  |  | >=0.6 | 15,287,903 | 89.19 | 1,241,370 | 77.28 |
|  |  | >=0.65 | 15,121,345 | 88.22 | 1,207,202 | 77.53 |
|  |  | >=0.7 | 14,935,134 | 87.13 | 1,166,424 | 77.82 |
|  |  | >=0.75 | 14,713,622 | 85.84 | 1,116,936 | 78.11 |
|  |  | >=0.8 | 14,440,645 | 84.25 | 1,053,710 | 78.44 |
|  |  | >=0.85 | 14,071,953 | 82.09 | 971,171 | 78.81 |
|  |  | >=0.9 | 13,489,541 | 78.7 | 852,078 | 79.2 |

**Table S7**. Permutation test of overlaps between predicted enhancers and external databases (empirical *p* < 0.001, N = 1000)

| Database | Genome coverage (%) | Observed overlap (bp) | Fold enrichment |
| --- | --- | --- | --- |
| DiseaseEnhancer | 0.061 | 1,149,942 | 1.706 |
| HACER | 10.15 | 237,448,869 | 1.331 |
| RAEdb | 0.48 | 34,153,011 | 2.190 |
| Vista | 0.058 | 1,137,305 | 2.002 |
| TiED | 0.63 | 15,706,823 | 2.504 |
| EI | 0.067 | 1,209,179 | 1.863 |
| EnhancerAtlas | 47.43 | 394,528,557 | 0.899 |
| HEDD | 13.1 | 304,125,372 | 1.938 |
| SEdb_TE | 62.79 | 24,203,082,239 | 1.605 |

**Table S8.** Whole genome prediction results: Length distribution of the predicted enhancer regions

| Length bins(bp) | prediction probability | | | | | | | | |
| --- | --- | --- | --- | --- | --- | --- | --- | --- | --- |
|  | **>=0.5** | **>=0.55** | **>=60** | **>=0.65** | **>=.70** | **>=0.75** | **>=0.8** | **>=0.85** | **>=0.9** |
| <500 | 1,227,484 | 1,223,620 | 1,213,264 | 1,196,039 | 1,170,954 | 1,136,572 | 1,086,640 | 1,015,760 | 905,870 |
| 501-1000 | 267,527 | 252,177 | 236,534 | 219,762 | 201,961 | 182,585 | 161,615 | 137,786 | 109,454 |
| 1001-1500 | 91,505 | 83,895 | 76,750 | 69,696 | 62,522 | 55,228 | 47,685 | 39,993 | 31,416 |
| 1501-5000 | 90,411 | 81,950 | 73,924 | 66,378 | 59,137 | 51,924 | 446,25 | 36,688 | 27,832 |
| 5001-10000 | 6,479 | 5,770 | 5,114 | 4,426 | 3,811 | 3,122 | 2,471 | 1,833 | 1,186 |
| 10001-20000 | 1,074 | 910 | 753 | 633 | 527 | 425 | 308 | 209 | 83 |
| 20001-30000 | 93 | 78 | 64 | 53 | 36 | 26 | 25 | 16 | 7 |
| 30001-40000 | 18 | 13 | 12 | 6 | 4 | 4 | 2 | 1 | 1 |
| >40000 | 4 | 4 | 3 | 3 | 3 | 2 | 0 | 0 | 0 |
| **Total** | **1,684,595** | **1,648,417** | **1,606,418** | **1,556,996** | **1,498,955** | **1,429,888** | **1,343,371** | **1,232,286** | **1,075,849** |

**Table S12**. Summary of LOF variants across LOR cutoffs, empirical p-values, and overlap with clinically significant variants (GWAS, ClinVar, eQTL).

| Cut-off applied | LOR cut-off value | #SNPs | Empirical p-value | #SNPs with reported clinical significance (GWAS/Clinvar/eQTL) |
| --- | --- | --- | --- | --- |
| 1σ | 1.758 | 5,289 | 0.085 | 13 |
| 2σ | 2.652 | 2,681 | 0.043 | 8 |
| 3σ | 3.547 | 1,665 | 0.027 | 4 |
| 4σ | 4.441 | 1,015 | 0.016 | 2 |
| 5σ | 5.335 | 537 | 0.009 | 1 |

**Table S13**. Summary of GOF variants across LOR cutoffs, empirical p-values, and overlap with clinically significant variants (GWAS, ClinVar, eQTL).

| Cut-off applied | LOR cut-off value | #SNPs | Emperical p-value | #SNPs with reported clinical significance (GWAS/Clinvar/eQTL) |
| --- | --- | --- | --- | --- |
| 1σ | -1.228 | 5,806 | 0.108 | 12 |
| 2σ | -1.768 | 1,917 | 0.036 | 6 |
| 3σ | -2.308 | 865 | 0.016 | 4 |
| 4σ | -2.848 | 497 | 0.009 | 2 |
| 5σ | -3.389 | 292 | 0.005 | 2 |

**Table S16**. Summary of potential gain-of-function (GOF) regulatory variants in non-enhancer regions across LOR cutoffs, empirical p-values, and overlap with clinically significant variants (GWAS, ClinVar, eQTL).

| Cut-off applied | LOR cut-off value | #SNPs | Emperical p-value | #SNPs with reported clinical significance (GWAS/Clinvar/eQTL) |
| --- | --- | --- | --- | --- |
| 1σ | -1.365 | 14,938 | 0.093 | 49 |
| 2σ | -2.002 | 5,464 | 0.034 | 10 |
| 3σ | -2.640 | 2,912 | 0.018 | 5 |
| 4σ | -3.278 | 1,753 | 0.011 | 4 |
| 5σ | -3.916 | 1,134 | 0.007 | 1 |
